# Investigation of interaction effects of biochars and silicon on growth and chemical composition of *Zea mays L.* in a Ni-polluted calcareous soil

**DOI:** 10.1038/s41598-023-47317-z

**Published:** 2023-11-15

**Authors:** Hamid Reza Boostani, Ailsa G. Hardie, Mahdi Najafi-Ghiri, Ehsan Bijanzadeh

**Affiliations:** 1 Department of Soil Science, College of Agriculture and Natural Resources of Darab, Shiraz University, Darab, Iran; 2 Department of Soil Science, Faculty of AgriSciences, Stellenbosch University, Private Bag X1, Matieland, 7602 South Africa; 3 Department of Soil Science, College of Agriculture and Natural Resources of Darab, Shiraz University, Darab, Iran; 4 Department of Agreoecology, College of Agriculture and Natural Resources of Darab, Shiraz University, Darab, Iran

**Keywords:** Environmental sciences, Agroecology

## Abstract

Biochars are cost effective, carbonaceous amendments that can immobilize potentially toxic elements (PTEs) in soils. Application of silicon (Si) has been shown to mitigate the effect of soil PTEs on plants. Studies on the interaction effects of Si and biochars in PTE-contaminated soils are limited. Thus, the aim of this study was to investigate the interaction effects of biochars, from sheep manure (SMB) and rice husk (RHB) produced at 300 and 500 °C, and 2 levels of Si (as sodium (Na) metasilicate), on growth and chemical composition of corn (*Zea mays*) in a nickel (Ni)-polluted calcareous soil. The combined application of Si and biochars significantly reduced soil available Ni (17–32%) and the corn shoot Ni concentrations (29–58%), associated with soil pH increase (r = 0.56–0.60, P < 0.01). Application of SMB resulted in greater soil pH increases compared to RHB, and increased soil electrical conductivity (EC) to saline levels, attributed to its higher ash content. Increasing Si application levels also increased soil pH and EC values. Application of all the biochars resulted in significant biomass increases, with RHB having the most positive effect. Despite the positive effect on soil Ni immobilization, the combined application of Si and biochars generally resulted in a decrease in corn shoot biomass yields compared to biochars alone. The biomass decrease was attributed to the significantly higher soil sodicity and pH in the combined treatments which resulted in suppression of macro and micronutrient uptake by the corn. Although the combination of biochar and Na metasilicate was effective at immobilizing soil Ni, future studies should rather employ other essential basic cation metasilicates.

## Introduction

Plants cannot complete their life cycle without sufficient amounts of soil nickel (Ni) present as it is regarded as an essential plant micronutrient. Nickel forms part of several important plant enzymes such as urease, superoxide dismutase, and hydrogenases, and thus plays an important role in N metabolism and free radical scavenging in plants^1^. However, Ni is also regarded as a potentially toxic element (PTE), with excessive soil concentrations leading to a significant decrease in plant growth by disturbing photosynthesis, root growth, enzymatic activity, and mineral nutrition^2^. Nickel demand is increasing worldwide due to its use in various industries such as electroplating, catalysis, electronics, pigments, coinage, batteries, ceramics and stainless steel. Waste disposal from these industries may lead to increases in the Ni concentration of soil environments^3^. Thus, in Ni-contaminated soils, it is of vital importance to apply eco-friendly, effectual, and cost-effective amendment materials to prevent Ni from entering into plants and the human food cycle.

Application of biochar in soils contaminated with PTEs has been demonstrated to be an inexpensive and efficacious procedure to reduce their bioavailability and translocation to plants^4^. Biochar is an organic, black, porous and stable material which obtained through pyrolysis process (200–900 °C) from organic wastes such as crop residues and manures^5^. Biochar is more cost effective to produce than activated carbon^4^, and is more economical and less disruptive to use than other physical or chemical means of soil decontamination^6^. Furthermore, biochar has multiple soil benefits such as enhancing soil nutrient and water holding capacity, and sequestering soil C^4–6^. The characteristics of biochar, including surface functional groups, high specific surface area, cation exchange capacity (CEC), and ash content result in a reduction of PTEs mobility in contaminated soils via processes such as adsorption, reduction, ion-exchange, and precipitation^6,7^. A large number of studies have confirmed the positive role of biochar application in increasing plant yield and nutrient uptake, and reducing PTE uptake in soils contaminated with PTEs^6–8^. Research has shown that the main mechanisms involved with biochar reducing Ni mobility in soils is precipitation and biochar surface metal sorption, and that biochars derived from rice residues are among the most effective^7^. Biochar not only promotes the immobilization of PTEs in the soil but also has been demonstrated to promote plant stress tolerance to PTEs^8,9^. Application of eucalyptus biochar (1–2% wt.) significantly reduced soil Ni availability and corn Ni uptake, and promoted corn growth (12–20%) in a slightly acid, Ni-contaminated clay loam soil^10^. Boostani et al.^11^ demonstrated that the application of licorice root and rice husk biochar (550 °C) at 2.5% wt. significantly enhanced spinach shoot dry matter by 15 and 60% respectively, in a Ni-contaminated calcareous soil, through the reduction of Ni uptake and plant stress related enzymes. Application of Casuarina biochar (4% wt.) in a highly contaminated soil caused a significant decrease in shoot Ni concentration of summer squash by 42.0% compared to control and increased shoot dry matter (SDM). Furthermore, soil organic matter (SOM), soil pH and electrical conductivity (EC) were significantly increased as influenced by Casuarina biochar addition^12^. In another study, Pescatore et al.^13^ observed that the application of pine biochar (mix of Douglas and Black Pine wood feedstocks) at two rates (0.8 and 1.6% wt.) to a PTEs-contaminated soil, SDM of berseem clover (*Trifolium alexandrinum* L.) was increased while shoot PTEs concentration decreased.

After oxygen, silicon (Si) is the most abundant element in the earth's crust. Monomeric and monosilicic (H_4_SiO_4_) forms of Si in soil are easily absorbed by plant roots. In PTE-contaminated soils, application of Si can improve the growth of plants by reducing PTE absorption, inhibiting PTE transfer from the roots to aerial parts, stimulating the antioxidant systems, and changing the structure of heavy metal gene expression^14^. Previously, an increase in the growth of plants such as wheat (*Triticum aestivum* L.), rice (*Oryza sativa* L.), peanut (*Arachis hypogaea* L.), and cotton (*Gossypium hirsutum* L.) in PTE-contaminated soils in the presence of Si has been reported^15^. Corn (*Zea mays* L.) is one of the most important crops in Iran, which, as a fodder for livestock production systems, makes a major contribution to providing human protein nutrition requirements. Maize consumption in Iran has increased from 2.5 million tons in 2001 to about 5 million tons in 2019^16^.

As both biochar and Si are effective at mitigating soil PTE availability and plant PTE-induced stress through different, but potentially complimentary mechanisms, we proposed to investigate their interaction effect. Currently, to our knowledge, no studies have been conducted on the interaction effects of Si with biochar in PTE-contaminated calcareous soils. Previous studies have only investigated the production of biochar-silicate composites on removing PTEs from solution. Deng et al.^17^ synthesized a novel pine sawdust hydrochar and magnesium silicate composite that was highly effective in removing zinc and copper from aqueous solutions. Similarly, Narasimharao et al.^18^ engineered a composite of orange peel waste biochar and magnesium silicate that was effective at the removal of hexavalent uranium ions from aqueous solution, especially compared to magnesium silicate. We hypothesized that soil Ni bioavailability, micro and macro-nutrients uptake by corn grown in a Ni-contaminated soil may vary by addition of biochar and different Si levels to the soil.

Therefore, the aim of this study was to investigate the interaction effects of biochars from different sources and Si on growth and chemical composition of corn, and selected characteristics of a Ni-polluted calcareous soil.

## Materials and methods

### Soil sampling, characterization and treatment

A surface soil sample (0–20 cm) was compositely collected from the agricultural fields of the College of Agriculture and Natural Resources of Darab, located in southern Iran (28°45′0.99″ N 54°26′52.14″ E, Elevation 1105 m). After air-drying and passing through a 2 mm sieve, the soil sample was subjected to routine analysis using various methods to determine its characteristics; soil texture analyzed by the hydrometer method^19^, pH of the saturated paste^20^, electrical conductivity (ECe) of saturated paste extract^21^, organic carbon (OC) determined by oxidation with chromic acid and titration with ferrous ammonium sulfate^22^, calcium carbonate equivalent (CCE) determined by neutralization with hydrochloric acid and titration with sodium hydroxide^23^, available phosphorous (P) extracted with sodium bicarbonate^24^, and available zinc, copper, manganese, iron and Ni extracted with diethylene triamine pentaacetic acid, DTPA^25^, with their elemental concentrations determined respectively using spectrophotometry and atomic absorption (PG 990, PG Instruments Ltd. UK). The physicochemical characteristics of the soil are given in Table 1. The soil was calcareous in nature with alkaline pH and low content of organic matter. Except for Zn, the content of other micronutrients were in the sufficient range for corn growth. Based on the soil EC value, it was in the category of non-saline soils.

**Table 1:** Some physicochemical characteristics of the soil before cultivation.

Sand (%)	58.00 (± 1.05)	Available K (mg kg^−1^)	251.00 (± 3.50)
Silt (%)	30.00 (± 0.25)	Available P (mg kg^−1^)	13.00 (± 0.02)
Clay (%)	12.00 (± 0.10)	CEC (cmol_(+)_kg^−1^)	11.70 (± 0.30)
Soil textural class	Sandy loam	Fe-DTPA (mg kg^−1^)	4.64 (± 0.10)
pH_(s)_	7.59 (± 0.04)	Mn-DTPA (mg kg^−1^)	12.30 (± 0.90)
EC (dS m^−1^)	2.60 (± 0.05)	Cu-DTPA (mg kg^−1^)	1.33 (± 0.10)
CCE (%)	55.00 (± 3.50)	Zn-DTPA (mg kg^−1^)	0.64 (± 0.20)
OM (%)	0.50 (± 0.08)	Ni-DTPA (mg kg^−1^)	0.39 (± 0.01)

*EC* electrical conductivity, *OM* organic matter, *CCE* calcium carbonate equivalent, *CEC* cation exchange capacity.

The procedure of Boostani et al.^26^ was used to contaminate 2 kg soil samples with Ni, using Ni(Cl)_2_ solution applied at 300 mg Ni kg^−1^ soil. Subsequently, soil samples were treated with Si using Na_2_SiO_3_ (sodium metasilicate) solution based on the experimental design (Section "Experimental design").

### Biochar production and its properties

Two raw organic materials from different sources (crop and livestock), namely, rice husk and sheep manure, were selected to produce biochars. After obtaining the feedstocks, they were air-dried and ground, and then placed in an oven for 24 h at 105 °C. The powdered biomass underwent slow pyrolysis in an electric muffle furnace (Shimifan, F47) at temperatures of 300 and 500 °C under limited oxygen conditions. The temperature was gradually increased from room temperature by 5 °C per minute until it reached the final temperature, which was maintained for 2 h to facilitate slow pyrolysis. The produced biochars were allowed to cool slowly and passed through a 0.5 mm sieve for uniformity.

The basic chemical properties of the produced biochars were determined as follows: pH was measured by adding 1.0 g of the sample to 20 mL of deionized (DI) water, shaking the suspension with a mechanical shaker at 40 rpm for 1 h, and then measuring the pH with a pH meter after equilibrating for 5 min^27^. Electrical conductivity (EC) was measured using a 1:20 biochar/DI water ratio after shaking for 30 min at 25 °C^28^. The percentage of carbon, nitrogen, and hydrogen in the biochar samples was determined using a CHN analyzer (ThermoFinnigan Flash EA 1112 Series). Cation exchange capacity (CEC) was measured using the ammonium acetate method^29^ by adding 20 mL of NH4OAC (1 M) at pH 7.0 to 4 g of biochar sample, shaking the mixture for 1 h, filtering it through Whatman 42 filter paper, and finally measuring the concentration of Ca, Mg, Na, and K in the filtrate. Total Ni, Fe, Mn, Cu and Zn was determined using atomic absorption (PG 990, PG Instruments Ltd. UK) after ashing at 550 °C and acid dissolution^30^, while total P was determined in the acid-digested ash fraction using the molybdate-ascorbic acid procedure by spectrophotometry at a wavelength of 460 nm^31^. To determine the ash content of the biochars, an open-top crucible was heated at 750 °C for 1 h. The moisture content of the biochars was determined by heating a 1.00 g biochar sample at 105 °C in an oven for 24 h. The content of O + S was calculated by subtracting the ash, moisture, C, N, and H from the total mass^27^. The physicochemical properties of the biochars are shown in Table 2.

**Table 2:** Some physical and chemical properties of the biochars.

	SMB3	SMB5	RHB3	RHB5
pH (1:20)	9.96 (± 0.03)	11.00 (± 0.02)	9.00 (± 0.04)	10.30 (± 0.03)
EC (1:20) (dS m^−1^)	3.94 (± 0.06)	4.28 (± 0.04)	0.84 (± 0.01)	1.17 (± 0.03)
CEC (cmol_+_ kg^−1^)	19.70 (± 0.8)	18.94 (± 0.65)	18.94 (± 0.35)	15.33 (± 0.40)
C (%)	25.40 (± 1.50)	31.80 (± 2.01)	45.00 (± 2.60)	50.00 (± 3.50)
H (%)	1.85 (± 0.09)	0.80 (± 0.01)	2.28 (± 0.07)	1.06 (± 0.05)
N (%)	2.10 (± 0.10)	1.57 (± 0.15)	1.30 (± 0.09)	1.10 (± 0.11)
P (%)	0.36 (± 0.01)	0.38 (± 0.03)	0.20 (± 0.01)	0.23 (± 0.01)
K (%)	2.36 (± 0.01)	2.47 (± 0.01)	0.81 (± 0.01)	1.02 (± 0.01)
Fe (mg kg^−1^)	1875.00 (± 10.50)	2019.00 (± 9.30)	207.00 (± 3.01)	358.00 (± 5.09)
Mn (mg kg^−1^)	236.00 (± 2.50)	241.00 (± 2.75)	105.00 (± 1.60)	139.00 (± 1.53)
Cu (mg kg^−1^)	20.10 (± 0.70)	20.80 (± 0.90)	1.50 (± 0.03)	2.80 (± 0.04)
Zn (mg kg^−1^)	52.10 (± 1.01)	60.70 (± 1.60)	18.20 (± 0.90)	18.50 (± 0.70)
Ni (mg kg^−1^)	3.00 (± 0.20)	15.40 (± 0.60)	Nd	Nd
Na (%)	0.73 (± 0.01)	0.77 (± 0.01)	0.06 (± 0.01)	0.07 (± 0.01)
Ca (%)	5.80 (± 0.08)	7.50 (± 0.05)	0.21 (± 0.01)	0.25 (± 0.01)
Moisture content (%)	1.91 (± 0.01)	1.82 (± 0.01)	2.65 (± 0.01)	2.37 (± 0.01)
Ash content (%)	53.80 (± 1.01)	60.00 (± 1.60)	34.20 (± 0.90)	44.80 (± 0.70)
H:C mole ratio	0.87 (± 0.01)	0.30 (± 0.01)	0.60 (± 0.01)	0.25 (± 0.01)
O + S:C mole ratio	0.44 (± 0.01)	0.09 (± 0.01)	0.24 (± 0.01)	0.01 (± 0.001)
C:N ratio	12.10 (± 0.10)	20.20 (± 0.12)	34.60 (± 0.15)	45.40 (± 0.16)

*SMB3* sheep manure biochar generated at 300 °C, *SMB5* sheep manure biochar generated at 500 °C, *RHB3* rice husk biochar produced at 300 °C, *RHB5* rice husk biochar produced at 500 °C, *CEC* cation exchange capacity, *EC* electrical conductivity, *Nd* non-detectable.

### Experimental design

A factorial experiment as completely randomized design was carried out with three replications under greenhouse conditions. The first factor was biochar treatment (with no biochar application (CL), sheep manure biochar produced at 300 °C (SMB3), sheep manure biochar produced at 500 °C (SMB5), rice husk biochar produced at 300 °C (RHB3), and rice husk biochar produced at 500 °C (RHB5), each at 3% (w/w)) and the second factor included Si levels (0 (Si_0_), 250 (Si_1_) and 500 (Si_2_) mg Si kg^−1^ soil). The experimental design is shown in Table 3.

**Table 3:** Experimental design of the present study.

	CL	SMB3	SMB5	RHB3	RHB5
Si_0_	CL + Si_0_	SMB3 + Si_0_	SMB5 + Si_0_	RHB3 + Si_0_	RHB5 + Si_0_
Si_1_	CL + Si_1_	SMB3 + Si_1_	SMB5 + Si_1_	RHB3 + Si_1_	RHB5 + Si_0_
Si_2_	CL + Si_2_	SMB3 + Si_2_	SMB5 + Si_2_	RHB3 + Si_2_	RHB5 + Si_0_

*CL* control, *SMB3* sheep manure biochar generated at 300 °C, *SMB5* sheep manure biochar generated at 500 °C, *RHB3* rice husk biochar produced at 300 °C, *RHB5* rice husk biochar produced at 500 °C, *Si*_*0*_ without Si application, *Si*_*1*_ addition of 250 mg Si kg^−1^ soil, *Si*_*2*_ addition of 500 mg Si kg^−1^ soil.

### Greenhouse experiment

Two kilograms of treated soil (with biochars and Si levels) according to the experimental design were transferred to plastic pots (45 experimental units), and then 6 seeds of corn (*Zea mays* L. 604) (prepared from Darab Agricultural Research Center) were planted in each of them at a depth of 2 cm. At the three-leaf stage, only 2 plants were kept in each pot. During the growth period, soil moisture was maintained at field capacity level using distilled water through the daily weighing of the pots. At the end of growth period (90 days), the aboveground parts of the plants were harvested. After washing with distilled water, the plant material was placed in an oven at 65 °C for 48 h and weighed immediately to measure shoot dry weight. The roots were then separated from the soil, and the soil was air-dried and passed through a 2 mm sieve. The soil pH and electrical conductivity (EC) were determined in saturated paste and saturated paste extract, respectively. The soil organic matter content was measured by oxidation with chromic acid and titration with ferrous ammonium sulfate^22^. Soil Ni availability was measured by DTPA solution (pH = 7.3)^25^. In order to measure the concentration of nutrients in the shoots, the material was ashed (550 °C for 2 h) and dissolved in 2 M HCl^10^. In the obtained extract, the content of Ni, Fe, Mn, Cu and Zn were measured by an atomic absorption spectroscopy (AAS) (PG 990, PG Instruments Ltd. UK). While the Na, K and Ca concentration were determined by a flame photometer (Corning 510, UK). The P content in the extract was measured by colormetric method (molybdate-ascorbic acid) at 460 nm wavelength on a spectrophotometer (SPECTRONIC 20D+). Nutrient uptake was calculated by multiplying the shoot dry matter yield with the nutrient concentration. It is noteworthy that the use of plant in the present study complies with international, national and/or institutional guidelines.

### Statistical analysis

The experiment was performed with three replicates. All the data were analysed using one-way ANOVA with MSTATC computer software package. Data obtained from the studies were subjected to Duncan's post-hoc test for comparing mean difference between treatments. All the statistical analyses were done at *p* ≤ 0.05 level of significance.

## Results and discussion

### Properties of the biochars

The maximum pH and EC values of the biochars were obtained in the SMB treatments, which increased with increasing pyrolysis temperature from 300 °C to 500 °C (Table 2). This is attributed to the higher content of ash (alkali salts) in the SMB biochars than those for the RHB biochars (Table 2). It is normal for plant-based biochars to contain lower amounts of dissolved solids than animal-waste based biochars^32^. The concentration of micronutrients (Fe, Mn, Cu and Zn), P and K in the biochars increased with pyrolysis temperature increase, and the highest content was found in the SMB (Table 2). The ash content of biochars is directly related to content of nutrients which are not volatized^33^ (Table 2). The CEC values of the biochars decreased with increasing pyrolysis temperature, with SMB3 having the highest value (19.70 cmol_+_ kg^−1^) (Table 2). This could be due to the reduction of surface functional groups such as carboxyl and phenol at high pyrolysis temperature, which are the main groups responsible for generating the CEC of biochars^34^.

With increasing pyrolysis temperature, the C content of the biochars were enhanced while, the H, O and N contents decreased (Table 2). The enhancement in C quantity at higher pyrolysis temperature coincides with the increasing degree of carbonization. While decreasing H and O amount is likely due to dehydration reactions, the decomposition of the oxygenated bonds, and the release of low molecular weight byproducts containing H and O^35^. Also, the volatilization of N compounds could be attributed to the decreased N content of the biochars at higher pyrolysis temperatures. The H:C and O:C mole ratios indicate the degree of aromaticity and polarity of the biochars, respectively^33^. The decreased H:C and O:C mole ratios with increasing the pyrolysis temperature (Table 2) indicates a better degree of carbonization of the biochars^35^. The Ni content of the RHB biochars was negligible, but the biochars produced from sheep manure contained a small amount of Ni (Table 2).

### Effects of biochars and Si application rates on the soil EC and pH

The main and interaction effects of treatments on the soil EC and pH values were significant (P < 0.05) (Table 4).

**Table 4:** Mean squares, F-values and P-values for the measured parameters in the present study.

	Mean square			F-value			P-value		
	Si	Biochar	Interaction	Si	Biochar	Interaction	Si	Biochar	Interaction
EC	0.866	7.344	0.040	176.24	1494.72	8.16	0.000	0.000	0.000
pH	0.470	0.049	0.016	198.81	20.57	6.56	0.000	0.000	0.000
Ni-DTPA	52.39	34.57	0.89	312.91	206.49	5.35	0.000	0.000	0.000
Ni-shoot	64.88	30.62	5.71	160.23	75.62	14.12	0.000	0.000	0.000
Shoot dry matter	0.21	0.72	0.078	365.08	1232.91	132.15	0.108	0.080	0.001
K-uptake	6.27	7.61	0.52	673.86	816.01	56.64	0.000	0.000	0.000
Ca-uptake	0.20	0.99	0.045	104.44	512.99	23.05	0.000	0.000	0.000
P-uptake	0.01	0.04	0.005	116.17	497.85	52.51	0.000	0.000	0.000
Fe-uptake	1859.88	979.92	269.52	235.22	123.93	34.08	0.000	0.000	0.000
Mn-Uptake	3315.29	7077.62	618.25	191.27	408.34	35.67	0.000	0.000	0.000
Cu-uptake	20.92	9.84	1.96	174.94	82.35	16.42	0.000	0.000	0.000
Zn-uptake	468.88	177.23	72.51	105.45	39.86	16.30	0.000	0.000	0.000

Addition of all the biochars caused a significant increase in the soil EC values. In particular, the SMB treatment resulted in EC values above 4 dS m^−1^ thus resulting in the treated soils to be classified as saline^36^. The minimum enhancement was observed in the RHB3 treatment by 21.1% (Table 5). As shown in Table 2, the EC and ash content of the RHB treatments were considerably lower than those of SMB treatments. Application of manure derived biochars with high ash content result in the greatest increases in soil salinity^34^. Additionally, in all the biochar treatments, application of Si rates from Si_0_ to Si_2_, caused a significant increase in the soil EC, with the greatest enhancement in the SMB5 + Si_2_ treatment by 17.8% (Table 5). The increase in soil EC values as a result of Si application rates is attributed to the high solubility of sodium metasilicate. Furthermore, the application of sodium metasilicate to the control soil (CL) without biochar, resulted in a significant pH increase (Table 5). The increase in soil pH is attributed to the dissolution of sodium metasilicate in water resulting in the hydrolysis of metasilicate ions to form monosilic acid, along with the formation of strong base sodium hydroxide^37^ (Eq. 1).

1 $${\text{2Na}}_{{2}} {\text{SiO}}_{{3}} + {\text{6H}}_{{2}} {\text{O}} \to {\text{2SiOH}}_{{4}} + {\text{4NaOH}}$$

**Table 5:** Effects of biochars and Si application rates on the soil EC (dS m^−1^) and pH after corn cultivation.

	CL	SMB3	SMB5	RHB3	RHB5	
EC						
Si_0_	2.55 (± 0.05)^k^	4.56 (± 0.06)^c^	4.25 (± 0.05)^d^	3.07 (± 0.03)^h^	3.45 (± 0.05)^g^	3.57 **C**
Si_1_	2.68 (± 0.09)^j^	4.71 (± 0.09)^b^	4.76 (± 0.06)^b^	3.15 (± 0.05)^h^	3.67 (± 0.03)^ef^	3.79 **B**
Si_2_	2.85 (± 0.05)^i^	5.07 (± 0.03)^a^	5.01 (± 0.19)^a^	3.56 (± 0.04)^fg^	3.79 (± 0.01)^e^	4.05 **A**
Mean	2.69 **E**	4.78 **A**	4.67 **B**	3.26 **D**	3.63 **C**	
pH						
Si_0_	7.69 (± 0.09)^j^	7.88 (± 0.02)^i^	7.96 (± 0.04)^gh^	7.73 (± 0.06)^j^	7.84 (± 0.05)^i^	7.82 **C**
Si_1_	8.07 (± 0.03)^de^	7.99 (± 0.01)^fg^	8.13 (± 0.02)^cd^	7.90 (± 0.05)^hi^	8.04 (± 0.06)^ef^	8.02 **B**
Si_2_	8.28 (± 0.02)^a^	8.17 (± 0.03)^bc^	8.22 (± 0.08)^ab^	8.05 (± 0.05)^ef^	8.16 (± 0.04)^bc^	8.18 **A**
Mean	8.01 **B**	8.01 **B**	8.10 **A**	7.89 **C**	8.01 **B**	

*CL* control, *SMB3* sheep manure biochar generated at 300 °C, *SMB5* sheep manure biochar generated at 500 °C, *RHB3* rice husk biochar produced at 300 °C, *RHB5* rice husk biochar produced at 500 °C, *Si*_*0*_ without Si application, *Si*_*1*_ addition of 250 mg Si kg^−1^ soil, *Si*_*2*_ addition of 500 mg Si kg^−1^ soil. Numbers followed by same letters in each column and rows, in each section, are not significantly (P < 0.05) different.

The interaction effects of the treatments showed that in the absence of sodium metasilicate (Si_0_), all the biochars except RHB3 significantly increased soil pH (Table 5). Whereas, at the Si_1_ level, the biochars produced at 300 °C caused a significant decrease in the soil pH values, and at the Si_2_ level, the pH values were significantly decreased by application of all the biochars except SMB5 (Table 5). It is likely that in the Si_1_ and Si_2_ treatments that the significant addition of salts to the by the biochars (Table 5) resulted in the displacement of exchangeable hydrogen ions present on the surface of the soil particles, resulting in a measured pH decrease. Furthermore, the magnitude of soil pH increase due metasilicate application in the CL treatment (absence of biochar) was much higher than the biochar treatments (Table 5). This could be due to the increase of soil buffering capacity as a result of biochar addition to the soil, as biochar contains acidic organic functional groups which can accept protons^32^.

### Soil Ni availability and shoot Ni concentration as affected by biochars and Si application rates

The main effects of treatments and their interactions on the soil Ni-DTPA and shoot Ni-concentration of corn were significant (P < 0.05) (Table 4). Generally, with increasing Si application rates from Si_0_ to Si_2_, the soil Ni-DTPA was significantly decreased (14.8%), and as a result, the concentration of Ni in the aerial parts of corn also decreased significantly (32.0%) (Table 6). In addition, application of all the biochar treatments caused a significant decrease in the content of soil Ni-DTPA and corn Ni-shoots (Table 6). The lowest concentrations of soil Ni-DTPA and corn Ni-shoots were associated with the combined treatment of SMB5 + Si_2_, with their concentrations decreasing by 32.4% and 57.2% compared to the control treatment (with no biochar and Si application), respectively. The SMB5 treatment had the highest pH and ash content among the biochars (Table 2) and resulted in the highest soil pH (Table 5). The Soil Ni-DTPA (r = -0.56, P < 0.01) and corn Ni-shoots (r = -0.60, P < 0.01) had a negative and significant correlation with soil pH. This confirms that one of the main reasons for decreasing the shoot Ni content and soil Ni-DTPA is rising soil pH as influenced by Si and biochar application. Several previous studies have also reported that the decline in soil Ni availability due to biochar application is associated with soil pH increase^7^. Ali et al.^38^ reported that the application of rice straw biochar (2% wt.) significantly reduced Ni availability in a contaminated, acid silty clay-loam soil. They observed a 129% increase in the least bioavailable residual soil Ni fraction which was associated with soil pH increase. Similarly, Boostani et al.^26^ found that the application of licorice root pulp and rice husk biochars (applied at 2.5% wt.) significantly reduced the soil Ni mobility factor (5–15%) and shoot Ni concentration of spinach (54–77%) in a contaminated, calcareous sandy loam soil. They also concluded soil pH increase due to application of biochars transformed soil Ni chemical fractions from bioavailable forms (soluble and exchangeable) to the residual fraction. The formation of insoluble Ni-silicates due to the combined application of silicate could also have contributed to decreased soil Ni-DTPA concentration^39^.

**Table 6:** Soil Ni availability (Ni-DTPA) (mg Ni kg^−1^ soil) and shoot Ni concentration (mg Ni kg^−1^ DM) as affected by biochars and Si application rates.

	CL	SMB3	SMB5	RHB3	RHB5	Mean
Ni-DTPA						
Si_0_	25.60 (± 0.03)^a^	24.20 (± 0.12)^b^	22.00 (± 0.35)^d^	22.30 (± 1.02)^cd^	20.40 (± 0.87)^f^	22.90 **A**
Si_1_	22.70 (± 0.07)^c^	21.20 (± 0.18)^e^	17.70 (± 0.10)^i^	19.20 (± 0.06)^g^	18.60 (± 0.06)^gh^	19.90 **B**
Si_2_	22.30 (± 0.02)^cd^	20.60 (± 0.19)^ef^	17.30 (± 0.05)^i^	18.70 (± 0.64)^gh^	18.40 (± 0.05)^h^	19.50 **B**
Mean	23.50 **A**	22.00 **B**	19.00 **D**	20.10 **C**	19.20 **D**	
Shoot Ni concentration						
Si_0_	10.40 (± 0.20)^a^	7.35 (± 0.15)^bc^	9.85 (± 0.05)^a^	7.55 (± 0.25)^bc^	7.65 (± 0.75)^b^	8.56 **A**
Si_1_	7.65 (± 0.05)^b^	6.90 (± 0.60)^bc^	6.60 (± 0.60)^cd^	7.05 (± 0.35)^bc^	7.35 (± 0.35)^bc^	7.11 **B**
Si_2_	7.20 (± 0.30)^bc^	5.05 (± 0.65)^ef^	4.45 (± 0.05)^f^	5.80 (± 0.70)^de^	6.60 (± 0.10)^cd^	5.82 **C**
Mean	8.41 **A**	6.43 **C**	6.96 **BC**	6.80 **BC**	7.20 **B**	

*CL* control, *SMB3* sheep manure biochar generated at 300 °C, *SMB5* sheep manure biochar generated at 500 °C, *RHB3* rice husk biochar produced at 300 °C, *RHB5* rice husk biochar produced at 500 °C, *Si*_*0*_ without Si application, *Si*_*1*_ addition of 250 mg Si kg^−1^ soil, *Si*_*2*_ addition of 500 mg Si kg^−1^ soil. Numbers followed by same letters in each column and rows, in each section, are not significantly (P < 0.05) different.

### Shoot dry matter of *Zea mays* L. as influenced by biochars and Si application rates

**Figure 1:**
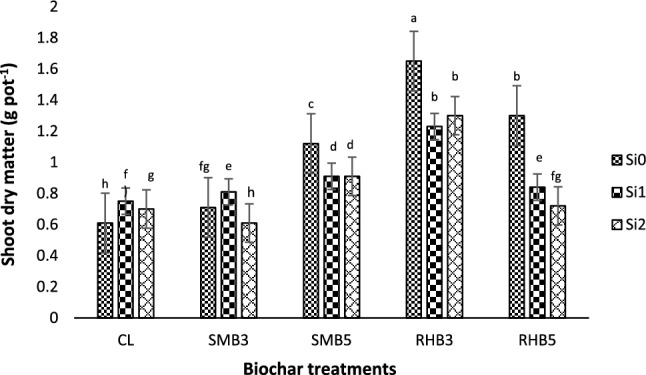
Interaction effects of biochars and silicon on shoot dry matter (g pot^−1^) of corn in a Ni-polluted calcareous soil. Notes: CL, control; SMB3, sheep manure biochar generated at 300 °C; SMB5, sheep manure biochar generated at 500 °C; RHB3, rice husk biochar produced at 300 °C; RHB5, rice husk biochar produced at 500 °C; Si_0_, without Si application; Si_1_, addition of 250 mg Si kg^−1^ soil; Si_2_, addition of 500 mg Si kg^−1^ soil. Numbers followed by same letters in each columns are not significantly (P < 0.05) different.

The main effects of biochars and Si application rates on the shoot dry matter of *Zea mays L.* were not statistically significant, while their interaction effects were significant (P < 0.05) (Table 4). The maximum and minimum amount of shoot dry matter was found in the combined treatments of RHB + Si_0_ (1.65 g pot^−1^) and Cl + Si_0_ (0.60 g pot^−1^), respectively (Fig. 1).

Application of all the biochar treatments at the Si_0_ and Si_1_ rates caused a significant increase in the shoot dry matter compared to the control (with no biochar application), while at the Si_2_ application level only SMB5 and RHB3 treatments increased it significantly (Fig. 1). The positive effect of the biochars on plant shoot biomass at the S_0_ and S_1_ is attributed to the decrease in soil Ni availability (Table 6), as well as the addition of macro and micronutrients (Table 2)^5,8^. Among the biochars, RHB3 had the lowest pH, EC and Na values (Table 2), which resulted in the lowest soil EC and pH values among the biochar treatments at the Si_2_ treatment level which could explain the higher biomass yields due to more favorable soil chemical conditions^36^. Whereas, the combined treatments of SMB5 and Si resulted in the greatest reduction of soil Ni availability (Table 6) which could explain the higher biomass yields in SMB5 + S_2_ treatment compared to control + Si_2_ treatment.

In the control treatment (with no biochar application), the shoot dry matter was significantly increased by application of Si rates from Si_0_ to Si_2_ (16.3%), whereas it was significantly decreased in all the biochar treatments (Fig. 1). This could be due to the considerable increase in soil EC of biochar and Si combined treatments (Table 5). Furthermore, there was a significant negative correlation between soil pH and shoot dry matter yield (r = − 0.41, P < 0.01) as affected by application of treatments. Excessive soil alkalinity promotes the formation of insoluble trace metal and P complexes leading to a decrease in bioavailability of these essential elements, ultimately resulting in plant nutrient deficiencies and stunted growth^33^. Furthermore, excessive soil Na concentrations result in nutrient antagonisms with other basic cations resulting in plant growth suppression^36^. In contrast to this study, Babu and Nagabovanalli^40^ reported that the total biomass of rice plant (grain + husk + straw + root) grown in a Cd-contaminated soil was significantly increased by addition of different levels of Si (0, 250 and 500 kg ha^−1^) as Ca-silicates (CaSiO_3_). They reported that it could be due to soil Cd stabilization caused by Ca-silicate-induced soil pH increase.

### Uptake of macro- and micro-nutrients by corn shoots in a Ni-polluted calcareous soil as affected by biochars and Si application rates

The main effects of Si application rates showed that with increasing soil Si levels from Si_0_ to Si_2_, the uptake of K, Ca and P by corn shoots were significantly decreased by 34.1%, 23.1% and 18.5%, respectively (Table 7). The decrease in the absorption of Ca and K nutrients as influenced by the increase in the level of Na-silicate application, is likely due to the increase in Na in the soil solution, and the competition of Na with Ca and K for absorption by the corn roots^36^. Furthermore, there was a significant and negative correlation between soil pH and P uptake by corn shoots (r = -0.36, P < 0.05). Application of Na-silicate levels caused a significant increase in soil pH values (Table 5). This can enhance the soil P fixation and thus, decreasing its bioavailability for the plant^36^. The main effects of biochars indicated that all the biochars caused a significant increase in the uptake of Ca, K and P by corn shoots, so that the highest increase was associated with the RHB3 treatment (Table 7). The RHB3 contained the least ash and soluble salts among the biochars (Table 2), thus it resulted in the smallest increases in soil EC and pH (Table 5). This explains why RHB3 resulted in the greatest increase in shoot dry matter (Fig. 1) and ultimately better absorption of nutrients such as P, K and Ca. The interaction effects of treatments showed that the combined treatment of RHB3 + Si_0_ had the highest uptake of K (5.19 mg pot^−1^), Ca (1.46 mg pot^−1^) and P (0.40 mg pot^−1^) by the plant shoots (Table 7).

**Table 7:** Uptake of K, Ca and P (mg pot^−1^) by corn shoots in a Ni-polluted soil as affected by biochars and Si application rates.

	CL	SMB3	SMB5	RHB3	RHB5	
K						
Si_0_	2.10 (± 0.01)^f^	2.76 (± 0.08)^e^	4.39 (± 0.01)^b^	5.19 (± 0.05)^a^	4.19 (± 0.01)^b^	3.72 **A**
Si_1_	2.05 (± 0.02)^f^	2.67 (± 0.01)^e^	3.16 (± 0.13)^d^	3.85 (± 0.01)^c^	2.67 (± 0.01)^e^	2.88 **B**
Si_2_	1.58 (± 0.07)^g^	2.02 (± 0.01)^f^	2.80 (± 0.03)^e^	3.90 (± 0.01)^c^	1.98 (± 0.10)^f^	2.45 **C**
Mean	1.91 **E**	2.49 **D**	3.45 **B**	4.31 **A**	2.95 **C**	
Ca						
Si_0_	0.45 (± 0.01) g	0.68 (± 0.02)^de^	1.03 (± 0.08)^b^	1.46 (± 0.03)^a^	1.10 (± 0.03)^b^	0.95 **A**
Si_1_	0.47 (± 0.01) g	0.85 (± 0.01)^c^	0.75 (± 0.03)^c-e^	1.45 (± 0.01)^a^	1.08 (± 0.01)^b^	0.92 **A**
Si_2_	0.49 (± 0.01) g	0.67 (± 0.02)^ef^	0.55 (± 0.01)^fg^	1.15 (± 0.01)^b^	0.81 (± 0.03)^cd^	0.73 **B**
Mean	0.47 **D**	0.74 **C**	0.78 **C**	1.36 **A**	1.00 **B**	
P						
Si_0_	0.15 (± 0.01)^h^	0.18 (± 0.01)^gh^	0.29 (± 0.01)^c^	0.40 (± 0.01)^a^	0.32 (± 0.02)^bc^	0.27 **A**
Si_1_	0.17 (± 0.01)^gh^	0.22 (± 0.02)^ef^	0.24 (± 0.01)^de^	0.31 (± 0.01)^bc^	0.20 (± 0.01)^fg^	0.23 **B**
Si_2_	0.17 (± 0.01)^gh^	0.17 (± 0.01)^gh^	0.26 (± 0.01)^d^	0.33 (± 0.01)^b^	0.18 (± 0.01)^gh^	0.22 **B**
Mean	0.17 **E**	0.19 **D**	0.26 **B**	0.35 **A**	0.24 **C**	

*CL* control, *SMB3* sheep manure biochar generated at 300 °C, *SMB5* sheep manure biochar generated at 500 °C, *RHB3* rice husk biochar produced at 300 °C, *RHB5* rice husk biochar produced at 500 °C, *Si*_*0*_ without Si application, *Si*_*1*_ addition of 250 mg Si kg^−1^ soil, *Si*_*2*_ addition of 500 mg Si kg^−1^ soil. Numbers followed by same letters in each column and rows, in each section, are not significantly (P < 0.05) different.

The main effects of treatments and their interactions were significant (P < 0.05) on the uptake of Fe, Mn, Cu and Zn by corn shoots (Table 4). The uptake of Fe (42.8%), Mn (32.6%), Cu (39.8%) and Zn (41.7%) were significantly decreased by application of Si rates from Si_0_ to Si_2_ (Table 8). This is attributed to the following two reasons: (1) the enhancement of soil pH as affected by Si application levels subsequently decreasing soil micronutrient bioavailability (Table 5). The uptake of Fe (− 0.57**), Mn (− 0.58**), Cu (− 0.60**) and Zn (− 0.49**) by corn shoots were negatively correlated with soil pH. (2) The increase of soil soluble Na concentration resulting in micronutrient antagonisms for plant uptake^36^.

**Table 8:** Uptake of Fe, Mn, Cu and Zn (µg pot^−1^) by corn shoots in a Ni-polluted soil as affected by biochars and Si application rates.

	CL	SMB3	SMB5	RHB3	RHB5	
Fe						
Si_0_	29.50 (± 0.75)^f–h^	36.20 (± 0.80)^d–f^	56.50 (± 3.97)^b^	78.10 (± 2.08)^a^	57.80 (± 1.45)^b^	51.60 **A**
Si_1_	34.20 (± 0.14)^ef^	34.90 (± 0.69)^ef^	40.90 (± 4.84)^de^	50.10 (± 1.76)^bc^	30.30 (± 1.87)^f–h^	38.10 **B**
Si_2_	31.60 (± 0.35)^fg^	25.00 (± 0.40)^gh^	22.40 (± 1.27)^h^	43.70 (± 1.19)^cd^	25.10 (± 1.25)^gh^	29.50 **C**
	31.80 **C**	32.10 **C**	39.90 **B**	57.30 **A**	37.70 **B**	
Mn						
Si_0_	48.60 (± 0.77)^f^	65.40 (± 3.18)^cd^	72.70 (± 3.82)^c^	145.00 (± 4.50)^a^	117.00 (± 6.16)^b^	89.60 **A**
Si_1_	54.90 (± 1.77)^d–f^	70.90 (± 2.20)^c^	55.10 (± 2.97)^d–f^	107.00 (± 2.55)^b^	64.20 (± 3.73)^c–e^	70.40 **B**
Si_2_	43.70 (± 2.25)^f^	52.70 (± 1.01)^ef^	43.60 (± 1.04)^f^	108.00 (± 1.75)^b^	53.80 (± 2.30)^d–f^	60.40 **C**
	49.10 **E**	63.00 **C**	57.10 **D**	120.00 **A**	78.20 **B**	
Cu						
Si_0_	3.05 (± 0.47)^e–g^	4.55 (± 0.54)^cd^	6.35 (± 0.28)^b^	7.83 (± 0.96)^a^	6.47 (± 0.03)^b^	5.65 **A**
Si_1_	3.27 (± 0.02)^e–g^	3.85 (± 0.15)^d–f^	3.43 (± 0.04)^e–g^	4.97 (± 0.38)^c^	3.99 (± 0.22)^c–e^	3.90 **B**
Si_2_	2.91 (± 0.08)^fg^	2.77 (± 0.07)^g^	3.23 (± 0.07)^e–g^	4.81 (± 0.20)^cd^	3.25 (± 0.11)^e–g^	3.40 **C**
	3.07 **D**	3.72 **C**	4.33 **B**	5.87 **A**	4.57 **B**	
Zn						
Si_0_	16.50 (± 2.75)^b–d^	17.70 (± 0.97)^bc^	30.20 (± 6.73)^a^	30.20 (± 0.85)^a^	33.70 (± 1.45)^a^	25.60 **A**
Si_1_	18.10 (± 0.88)^bc^	13.20 (± 0.32)^c–e^	21.50 (± 0.78)^b^	21.40 (± 1.43)^b^	13.50 (± 0.55)^c–e^	17.50 **B**
Si_2_	16.30 (± 1.98)^b–d^	9.70 (± 0.20)^e^	16.20 (± 1.29)^b–d^	22.40 (± 0.08)^b^	10.10 (± 0.66)^de^	14.90 **C**
	17.00 **B**	13.50 **C**	22.60 **A**	24.60 **A**	19.10 **B**	

*CL* control, *SMB3* sheep manure biochar generated at 300 °C, *SMB5* sheep manure biochar generated at 500 °C, *RHB3* rice husk biochar produced at 300 °C, *RHB5* rice husk biochar produced at 500 °C, *Si*_*0*_ without Si application, *Si*_*1*_ addition of 250 mg Si kg^−1^ soil, *Si*_*2*_ addition of 500 mg Si kg^−1^ soil. Numbers followed by same letters in each column and rows, in each section, are not significantly (P < 0.05) different.

Application of all the biochar treatments caused a significant increase in the uptake Fe, Mn, Cu and Zn by corn shoots compared to the control, with RHB3 treatment having the greatest uptake (Table 8). The biochars used in this research contained significant amounts of the micronutrients, Fe, Mn, Cu, and Zn (Table 2), which could increase their absorption by plant roots. Although RHB3 contained the least micronutrients (Table 2), it enhanced micronutrient uptake to the greatest extent likely due to the resultant lower soil pH and EC values (Table 5) as previously discussed. The interaction effects of treatments showed that the combined treatment of RHB3 + Si_0_ had the highest uptake of Fe (78.1 µg pot^−1^), Mn (145.00 µg pot^−1^), Cu (7.83 µg pot^−1^) and Zn (30.20 µg pot^−1^) by the plant shoots (Table 8). Rehman et al.^10^ found that the application of eucalyptus wood biochar (2% wt.) to a Ni polluted soil significantly enhanced Fe, Mn, Cu and Zn uptake and suppressed Ni uptake by corn. They attributed this to a reduction in Ni bioavailability in the soil (61% reduction), as Ni is antagonistic to essential trace metal cation uptake by plants. The increase of the nutrients absorption by the plant as a result of adding biochars to the soil may also be due to the enhancement of plant growth, enhanced soil microbial activity, or the release of nutrients from the biochars during the growing season^41^.

## Conclusions

The combined application of Si (Na metasilicate) and biochars significantly reduced soil available Ni and the corn shoot Ni concentrations, which was related to soil pH increase. However, the combined application of Si and biochars generally resulted in a decrease in corn shoot biomass yield compared to biochars alone. The decreased corn yield in the combined treatments was attributed to the significantly higher soil pH and Na content, leading to suppression of macro and micronutrient uptake by the corn. Although combined application of biochars and silicate are highly effective in reducing the bioavailability of Ni in contaminated soils, future studies investigating the combined effect of Si and biochars should rather make use of Ca, or K silicates due to the antagonistic effects of Na on crop nutrient uptake.

## Data Availability

The datasets used and/or analyzed during the current research are available from the corresponding author on request.
